# Integrative Transcriptomic and Functional Analysis of PKMYT1 Reveals a Potential Therapeutic Target in Chronic Lymphocytic Leukemia

**DOI:** 10.1002/hon.70208

**Published:** 2026-06-06

**Authors:** Elizabete Cristina Iseke Bispo, Cláudia de Souza Lima Pontes, Jennifer Martins do Nascimento, Fábio Wilson de Lima Alves, Juliana Lott de Carvalho, Antônio Roberto Lucena‐Araujo, Felipe Saldanha‐Araujo

**Affiliations:** ^1^ Laboratório de Hematologia e Células‐Tronco Faculdade de Ciências da Saúde Universidade de Brasília Brasília Distrito Federal Brasil; ^2^ Laboratório de Biociências Faculdade de Medicina Universidade de Brasília Brasília Distrito Federal Brasil; ^3^ Laboratório de Hematologia Centro de Biociências Universidade Federal de Pernambuco Recife Pernambuco Brasil

**Keywords:** cell cycle regulation, chronic lymphocytic leukemia, complex karyotype, genomic instability, PKMYT1, therapeutic target

## Abstract

Chronic lymphocytic leukemia (CLL) is a clinically and molecularly heterogeneous disease. PKMYT1, a G2/M cell cycle kinase, has been implicated in tumor progression in several cancers, but its role in CLL remains unclear. We evaluated *PKMYT1* expression in primary CLL samples and analyzed associations with cytogenetic features and clinical parameters. *PKMYT1* expression was heterogeneous and correlated with adverse features, including complex karyotype and elevated leukocyte counts. Comparative transcriptomic analyses between high‐ and low‐expression groups revealed enrichment of pathways related to chromatin remodeling, DNA repair, and mitotic regulation. In MEC1 cells, pharmacological PKMYT1 inhibition significantly reduced cancer cell viability. PCR analysis showed upregulation of *TP53, CASP3, BAK, GSDMD, BCL2, CASP1, IL1β, RIPK1, and RIPK3*, indicating activation of apoptotic, inflammasome‐associated, and necroptotic pathways. Collectively, these findings demonstrate that *PKMYT1* is heterogeneously expressed in CLL, associated with adverse cytogenetic and clinical features, and critical for cell survival, highlighting its potential as a therapeutic target.

## 1 Introduction

Chronic lymphocytic leukemia (CLL) is a clonal malignancy of CD5^+^ B cells and represents the most common form of adult leukemia, particularly in aging populations worldwide. The clinical course of CLL is highly heterogeneous, with some patients remaining asymptomatic for years while others experience rapid disease progression, therapeutic resistance, and poor survival outcomes. This variability is largely influenced by underlying genomic alterations and disruptions in key regulatory pathways [1].

It is well established that genomic instability plays a central role in CLL pathogenesis and progression, driving the accumulation of chromosomal aberrations and subclonal evolution. However, the molecular mechanisms that govern the maintenance genome integrity in CLL cells remain incompletely understood [2, 3].

Among the pathways involved in genomic surveillance, the DNA damage response (DDR) and cell cycle checkpoints are critical for preventing the propagation of damaged DNA. The kinases WEE1 and PKMYT1 act as negative regulators of CDK1 activity, enforcing the G2/M checkpoint to allow time for DNA repair. While WEE1 has been extensively studied in hematologic malignancies and is currently under clinical investigation as a therapeutic target in acute myeloid leukemia and lymphomas [4, 5], the functional relevance of PKMYT1 in these contexts remains poorly defined.

PKMYT1 phosphorylates CDK1 at threonine 14 and tyrosine 15, playing a non‐redundant role in the regulation of mitotic entry [6]. Despite its involvement in cell cycle control and DDR, few studies have addressed its role in B‐cell malignancies, and no comprehensive evaluation has been performed in CLL to date.

In this study, we investigated *PKMYT1* expression in CLL and its associations with cytogenetic alterations, clinical parameters, and patient outcomes. We also performed functional enrichment analyses to explore transcriptional programs linked to *PKMYT1* expression and assess potential biological roles in genome maintenance and disease progression. Finally, we evaluated the effects of pharmacologic PKMYT1 inhibition on the viability of CLL cells in vitro.

## 2 Methods

### 2.1 Sample Collection

Peripheral blood samples were collected from 55 consecutive patients with chronic lymphocytic leukemia (CLL) who were diagnosed according to standard immunophenotypic criteria and treated at the University Hospital of the Medical School of Ribeirão Preto, University of São Paulo (Brazil). At the time of sample collection, patients were undergoing chemotherapy, as previously described [7]. In addition, peripheral blood samples from 10 age‐matched hematologically healthy donors were obtained as controls. The study was conducted in accordance with the Declaration of Helsinki and was approved by the local Ethics Committee. Written informed consent was obtained from all participants.

### 2.2 Sample Processing, Immunophenotyping, and Cytogenetic Characterization

Peripheral blood samples from CLL patients and healthy donors were processed for the isolation of peripheral blood mononuclear cells (PBMCs) using Ficoll‐Paque PLUS density gradient centrifugation (Amersham Biosciences). CD19+ B‐cells were subsequently enriched by immunomagnetic selection using anti‐CD19‐conjugated magnetic beads, according to the manufacturer's protocol (Miltenyi Biotec). The purity of isolated B‐cells exceeded 90%, as confirmed by flow cytometry. Immunophenotypic analysis of the CD19+CD5+ population was carried out to determine ZAP‐70 expression using a PE‐conjugated anti‐ZAP‐70 antibody (Dako), with isotype controls applied to define positivity (cut‐off: 20%). Flow cytometry acquisition was performed on a FACSCalibur cytometer, and data were analyzed using Cell Quest software (BD Biosciences). For cytogenetic analysis, PBMCs were cultured in RPMI 1640 medium supplemented with 20% fetal calf serum, interleukin‐2 (IL‐2), and CpG‐oligonucleotide DSP30 (TIBMolBiol) for 72 h. Metaphase arrest was induced with colcemid, and at least 20 metaphases per sample were evaluated by standard G‐banding. Complex karyotype was defined as the presence of more than three chromosomal abnormalities (≥ 3), according to ISCN 2016 [ISCN 2016: An International System for Human Cytogenetic Nomenclature, CT, 2016].

### 2.3 Real‐Time PCR (RT‐PCR)

Total RNA was extracted from purified CD19^+^ cells using TRIzol reagent (Invitrogen), according to the manufacturer's instructions. cDNA was synthesized from 1 μg of total RNA using the High Capacity cDNA Reverse Transcription Kit (Thermo Fisher Scientific), with random primers. Quantitative PCR was performed using the GoTaq qPCR Master Mix (Promega) on a QuantStudio Real‐Time PCR System (Thermo Fisher Scientific). In primary samples, the expression of *PKMYT1* was evaluated. In MEC1 cells treated with the PKMYT1 inhibitor at the IC_50_ dose or left untreated (control), the expression of genes associated with different cell death pathways was assessed, including *BAX*, *TP53*, *CASP3*, *BAK*, *CASP1*, *GSDMD*, *GSDME*, *BCL2*, *IL1β*, *RIPK1*, and *RIPK3*. Primer sequences used for amplification are available from the authors upon reasonable request.

All reactions were carried out in duplicate in a final volume of 20 μL. *ACTB* and *GAPDH* were used as the endogenous control. Relative gene expression levels were calculated by the 2^−ΔΔCt^ method, using the average ΔCt of healthy donor samples (for primary cells) or untreated MEC1 cells (for cell line experiments) as the calibrator.

### 2.4 In Silico Analysis of Gene Expression, Pathway Enrichment, and Survival Association

*PKMYT1* expression was initially assessed using the BloodSpot transcriptomic platform (https://www.fobinf.com), which integrates multiple hematopoietic datasets. Expression data from CLL samples (*n* = 448) and normal bone marrow (BM) cells (*n* = 73), derived from the MILE (Microarray Innovations in Leukemia) study [8], were used to evaluate differences between malignant and non‐malignant hematopoietic tissues.

Additional in silico analyses were performed using RNA‐seq data from a CLL cohort published by the Broad Institute [9], available via the cBioPortal for Cancer Genomics (https://www.cbioportal.org/). Patients were stratified into high and low expression groups based on the median mRNA levels of *PKMYT1*. Differentially expressed genes (DEGs) between groups were identified using cBioPortal's built‐in analysis tools, under default statistical parameters.

To prioritize genes for enrichment analysis, DEGs were ranked using a custom score calculated as the product of the log_2_ fold change (log_2_FC) and the log_10_‐transformed false discovery rate (FDR), which integrates both the magnitude and statistical significance of differential expression. Heatmaps representing the top 20 upregulated and top 20 downregulated genes (based on mean gene expression) were generated using the Morpheus platform (https://software.broadinstitute.org/morpheus).

Two complementary functional enrichment strategies were employed to interrogate biological pathways associated with *PKMYT1* expression. First, an Over‐Representation Analysis (ORA) was performed using DAVID (Database for Annotation, Visualization and Integrated Discovery), focusing on Gene Ontology (GO) terms across the Biological Process, Cellular Component, and Molecular Function categories. Second, Gene Set Enrichment Analysis (GSEA) was carried out using the GSEA desktop application (https://www.gsea‐msigdb.org/) and the Molecular Signatures Database (MSigDB), including the Hallmark, KEGG, and Reactome gene set collections [10, 11]. GSEA was run with 1000 permutations and the “No Collapse” setting enabled to preserve probe‐level identifiers.

Survival analyses were performed directly on cBioPortal by comparing overall survival between high and low expression groups for *PKMYT1*. Kaplan–Meier plots were generated, and statistical significance was assessed using the log‐rank test, as implemented on the platform.

### 2.5 PKMYT1 Chemical Inhibition, Cell Viability Assay (MTT), and Apoptosis Analysis by Flow Cytometry

The CLL cell line MEC1 was kindly provided by Prof. Rodrigo Alexandre Panepucci (Fundação Hemocentro de Ribeirão Preto—FUNDHERP, Brazil) and maintained under standard culture conditions. To assess the cytotoxic effects of GSK 1520489A (MedChemExpress), 50,000 cells per well were seeded in 96‐well plates and treated with increasing concentrations of each compound, ranging from 0 to 20,000 nM. After 48 h of drug exposure, cell viability was evaluated by MTT assay (Sigma Aldrich). MTT reagent (3‐(4,5‐dimethylthiazol‐2‐yl)‐2,5‐diphenyltetrazolium bromide) was added to each well and incubated for 4 h at 37°C. The resulting formazan crystals were solubilized, and absorbance was measured at 570 nm using a Multiskan FC microplate reader (Thermo Fisher Scientific). Parallel assays were performed using PBMCs from healthy donors under the same conditions, to assess compound toxicity in non‐malignant cells. The IC_50_ values were determined by nonlinear regression analysis. The IC_50_ values was determined by nonlinear regression analysis.

For apoptosis analysis, cells were subjected to Annexin V/propidium iodide (PI) staining and analyzed by flow cytometry, following the same experimental design used in the MTT assay. Briefly, at the end of the experiment, cells were harvested, washed twice with cold PBS, and resuspended in a binding buffer. Cells were then incubated with Annexin V‐APC and PI for 15 min at room temperature in the dark. Samples were analyzed using a FACSCalibur flow cytometer (BD Biosciences), and data were processed with FlowJo software.

### 2.6 Statistical Analysis

All statistical analyses were performed using GraphPad Prism 7 (GraphPad Software, San Diego, CA, USA) and the analytical tools embedded in cBioPortal, where applicable. Continuous variables were compared using the Mann–Whitney *U* test or Kruskal–Wallis test with Dunn's post hoc test, depending on the number of groups and data distribution. Categorical variables were compared using Fisher's exact test or the Chi‐square test, as appropriate. A *p*‐value < 0.05 was considered statistically significant. Survival analyses were conducted using Kaplan–Meier curves, with differences between groups assessed by the log‐rank test.

## 3 Results

**FIGURE 1:**
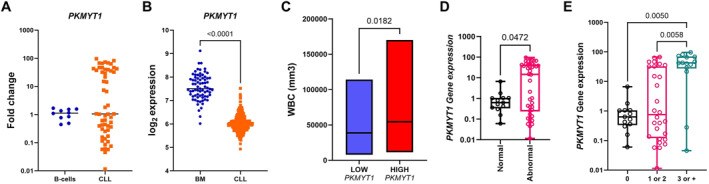
PKMYT1 expression and its association with clinical and cytogenetic features in CLL. (A) *PKMYT1* mRNA levels in CLL samples (*n* = 55) compared to healthy donor B‐cells (*n* = 10), as determined by quantitative real‐time PCR. (B) *PKMYT1* expression in CLL (*n* = 448) and normal bone marrow (BM) cells (*n* = 73), analyzed using the BloodSpot transcriptomic platform. (C) Comparison of white blood cell (WBC) counts between *PKMYT1*‐high and *PKMYT1*‐low CLL samples. (D, E) Associations between *PKMYT1* expression and presence of chromosomal alterations and number of acquired chromosomal alterations. Horizontal bars represent median values. Statistical comparisons were performed using the Mann–Whitney *U* test (for two‐group comparisons) and Kruskal–Wallis test followed by Dunn's post hoc test (for multiple groups).

Using data mining of the BloodSpot database, we observed that *PKMYT1* expression was significantly decreased in CLL samples compared to healthy bone marrow controls (*p* < 0.0001). In our cohort, however, *PKMYT1* expression did not significantly differ from that in B‐cells from healthy donors (Figure 1A, B). Despite the lack of significant difference in *PKMYT1* expression between our cohort and healthy donor B‐cells, we observed marked heterogeneity among the CLL samples. Therefore, we stratified them into low (≤ 1.05‐fold) and high (> 1.05‐fold) expression groups based on the median expression level.

Clinical and baseline characteristics of the patients included in our cohort are summarized in Table 1. Interestingly, patients with high *PKMYT1* expression exhibited significantly higher leukocyte counts compared to those with low expression (*p* = 0.01; Figure 1C). Moreover, individuals with karyotypic abnormalities showed elevated PKMYT1 expression levels (*p* = 0.04), with a particularly significant increase observed in patients harboring complex karyotypes, defined as the presence of more than three chromosomal abnormalities (≥ 3) (*p* = 0.002; Figure 1D–E). In this context, the association was primarily observed at the level of overall karyotypic complexity; the size of our cohort did not allow a reliable assessment of associations with individual chromosomal lesions.

**TABLE 1:** Clinical and baseline characteristics of the study population.

Characteristics	All patients (%)	PKMYT1		*p*
		Low	High	
Age, *y*				0.11
Median (range)	65 (43, 85)	59 (43, 85)	66 (43, 80)	
Sex				0.43
Female	22 (40)	12 (42.8)	10 (37.0)	
Male	33 (60)	16 (57.2)	17 (63.0)	
Binet				0.46
A	33 (60)	19 (67.9)	14 (51.9)	
B	8 (14.5)	3 (10.7)	5 (18.5)	
C	14 (25.5)	6 (21.4)	8 (29.6)	
Platelets				0.34
Median (range), X10^9^/L	141 (8.7, 312)	145 (8.7, 284)	137 (15, 312)	
WBC				0.25
Median (range), X10^9^/L	45.3 (7.8, 170.1)	38.7 (7.8, 114.2)	54.3 (11.3, 170.1)	
ZAP‐70^a^				0.34
Negative	17 (31.5)	10 (35.7)	7 (26.9)	
Positive	37 (68.5)	18 (64.3)	19 (73.1)	
Karyotype				**0.03**
Normal	13 (23.6)	10 (35.7)	3 (11.1)	
Abnormal	42 (76.4)	18 (64.3)	24 (88.9)	
Number of karyotypic changes				**0.003**
0	13 (23.6)	10 (35.7)	3 (11.2)	
1 or 2	28 (51)	16 (57.1)	12 (44.4)	
3 or more	14 (25.4)	2 (7.2)	12 (44.4)	

*Note:* Values are shown as number (percentage) or median (range), depending on the row headings. Statistically significant differences are indicated in bold.

^a^ Missing values were excluded from the statistical analysis.

**FIGURE 2:**
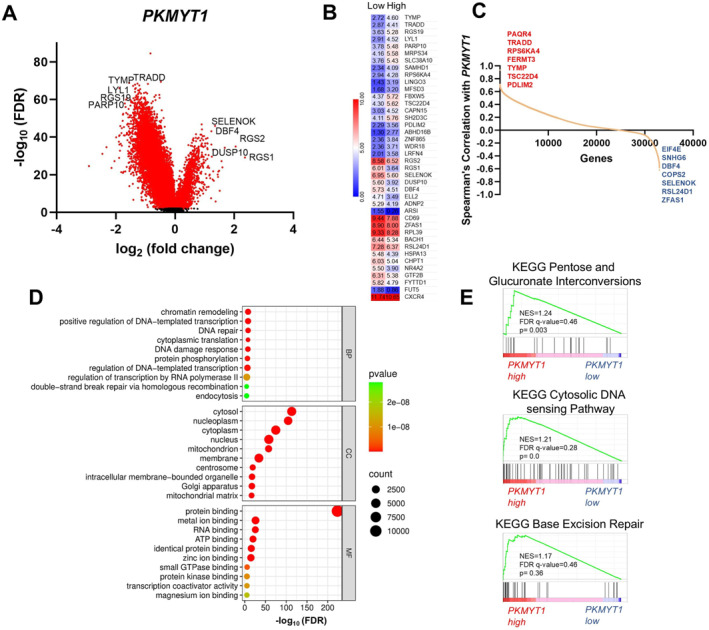
Integrated transcriptomic and functional analyses of *PKMYT1* expression in CLL. (A) Volcano plot showing differentially expressed genes (DEGs) in CLL samples stratified by *PKMYT1* expression levels. (B) Heatmap displaying the 20 most upregulated and 20 most downregulated genes in *PKMYT1*‐high versus PKMYT1‐low CLL samples. Values represent mean log_2_ expression for each group. (C) Correlation analysis identifying the seven genes most positively and negatively associated with *PKMYT1* expression, based on Spearman's correlation coefficients. (D) Gene Ontology (GO) enrichment analysis of DEGs showing the top 10 significantly enriched terms across Biological Process (BP), Cellular Component (CC), and Molecular Function (MF) categories. Enrichment is represented as –log_10_ FDR. (E) GSEA highlighting pathways and processes associated with *PKMYT1* expression. Normalized enrichment scores (NES), FDR q‐values, and *p*‐values are shown. This figure includes data derived from the KEGG database (https://www.kegg.jp/).

To further investigate the biological implications of *PKMYT1* expression in CLL, we initially compared cases with high versus low *PKMYT1* expression to identify differentially expressed genes (DEGs) and, subsequently, analyzed for pathway enrichment, using over‐representation analysis (ORA) and gene set enrichment analysis (GSEA). ORA, performed using DAVID, revealed significant enrichment in biological processes related to chromatin remodeling, DNA repair, DNA damage response, and transcriptional regulation. Enrichment of cellular components such as the centrosome, along with molecular functions including RNA binding, ATP binding, and protein kinase binding, suggests that transcriptional programs associated with *PKMYT1* involve pathways linked to genome maintenance, RNA processing, and signal transduction (Figure 2A–D).

In parallel, GSEA identified several gene sets with nominal enrichment across the Hallmark, KEGG, and Reactome databases. Although none reached statistical significance after multiple testing correction (FDR < 0.25), several top‐ranked sets, including those related to DNA replication, base excision repair, telomere maintenance, and metabolic processes, showed moderate normalized enrichment scores (NES) and nominal *p*‐values < 0.05 (Figure 2E).

**FIGURE 3:**
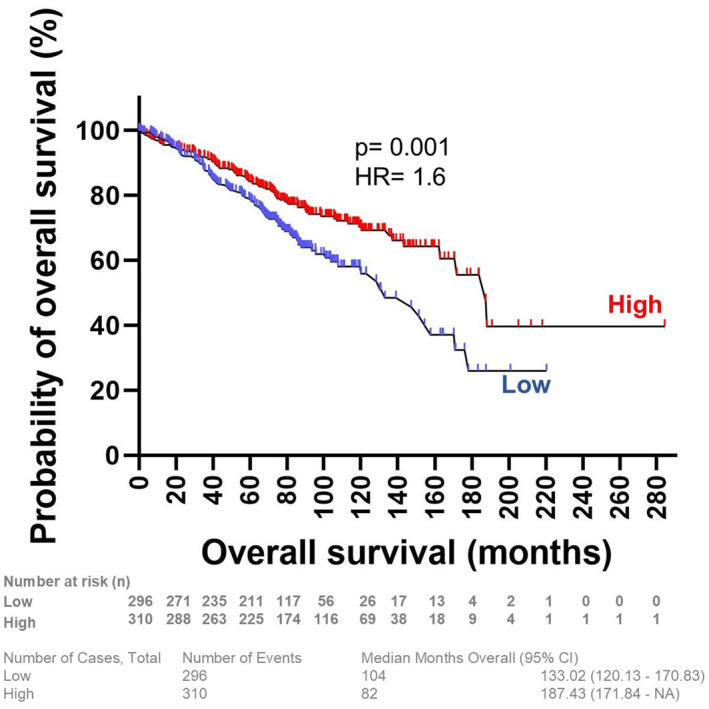
Survival analysis based on PKMYT1 expression in CLL patients. Kaplan–Meier curves depicting OS in CLL patients (*n* = 606) stratified by *PKMYT1* mRNA expression levels. Patients with low *PKMYT1* expression (*n* = 296) had a median OS of 133 months (95% CI: 120–170), with 104 death events, whereas those with high *PKMYT1* expression (*n* = 310) exhibited a significantly longer median OS of 187 months (95% CI: 171–not reached), with 82 events. Statistical comparison was performed using the log‐rank test.

Given the potential clinical relevance of these transcriptional alterations, we next evaluated the prognostic impact of PKMYT1 expression in CLL. Survival analysis of 606 patients showed that those with low *PKMYT1* expression (*n* = 296) had a median overall survival (OS) of 133 months (95% CI: 120–170), with 104 events. In contrast, patients with high *PKMYT1* expression (*n* = 310) exhibited significantly longer median OS of 187 months (95% CI: 171–not reached), with 82 events (deaths) (*p* = 0.001; Figure 3).

**FIGURE 4:**
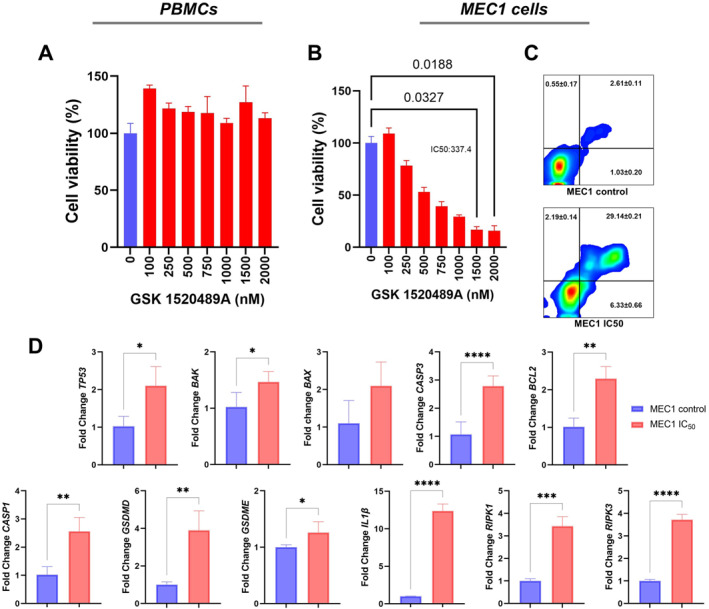
Impact of pharmacological PKMYT1 inhibition on PBMC and MEC1 cell viability and apoptosis (A) MTT assay in peripheral blood mononuclear cells (PBMCs) treated with GSK1520489 showing no significant cytotoxicity. (B) Dose–response viability assay in MEC1 cells treated with the PKMYT1 inhibitor GSK1520489, assessed by MTT. (C) Representative Annexin V/PI dot plots of untreated and GSK1520489‐treated MEC1 cells, indicating increased apoptosis upon treatment. (D) Relative mRNA expression levels of genes associated with cell death pathways in MEC1 cells treated with the PKMYT1 inhibitor GSK1520489 at the IC_50_ concentration, assessed by RT–qPCR. Expression of *TP53, BAX, BAK, CASP3, BCL2, CASP1, GSDMD, IL1B, RIPK1, and RIPK3* was evaluated and analyzed using the 2^−ΔΔCt^ method, with untreated cells used as calibrator. Data are presented as mean ± standard deviation (SD).

To further explore the functional role of PKMYT1 in CLL, we pharmacologically inhibited PKMYT1 in MEC1 cells using the inhibitor GSK 1520489A. This intervention led to a marked reduction in cell viability, indicating that CLL cells may rely on PKMYT1 activity for survival. Consistently, flow cytometry analysis using Annexin V/propidium iodide staining revealed a significant induction of apoptosis following PKMYT1 inhibition. Importantly, treatment with the same inhibitor did not affect the viability of PBMCs isolated from healthy blood donors, as assessed by MTT assay (Figure 4A–C).

To further characterize the cell death mechanisms triggered by PKMYT1 inhibition, we evaluated the expression of genes associated with apoptosis and inflammatory cell death pathways in MEC1 cells treated with the IC_50_ dose of the inhibitor GSK 1520489A. RT–qPCR analysis revealed significant upregulation of *TP53* (*p* = 0.01), *CASP3* (*p* < 0.0001), and *BAK* (*p* = 0.03), supporting activation of apoptotic signaling. In addition, genes related to inflammatory cell death pathways were also increased, including *GSDMD* (*p* = 0.004), *CASP1* (*p* = 0.004), *IL1β* (*p* < 0.0001), *RIPK1* (*p* = 0.0003), and *RIPK3* (*p* < 0.0001). Interestingly, expression of *BCL2* was also elevated (*p* = 0.002) in inhibitor‐treated cells (Figure 4D). Together, these findings suggest that PKMYT1 inhibition activates apoptotic signaling while also modulating genes involved in inflammatory cell death pathways.

## 4 Discussion

In CLL, most molecular studies have focused on canonical DDR genes, such as TP53 and ATM [12, 13, 14], while the potential contribution of mitotic kinases to genomic instability and disease progression remains largely underexplored. Although PKMYT1 is a key regulator of the G2/M checkpoint, its role in CLL has not been systematically investigated. Here, we combined transcriptomic analyses of patient‐derived samples with integrative bioinformatic profiling of public datasets and in vitro functional assays to characterize *PKMYT1* expression, its associated transcriptional programs, and potential implications for CLL biology.

Interestingly, *PKMYT1* expression differed depending on the dataset examined. In the BloodSpot database, expression was reduced in CLL samples compared to bulk bone marrow controls, whereas no significant difference was observed between CLL samples and normal B‐cells in our local cohort. This discrepancy likely reflects differences in the composition of control populations, as BloodSpot includes mixed bone marrow cells while our cohort used purified mature B‐cells. These observations highlight the heterogeneity of *PKMYT1* expression in CLL and underscore the importance of context when interpreting transcriptomic data. Notably, *PKMYT1* is frequently overexpressed in several cancers, including osteosarcoma and pancreatic ductal adenocarcinoma, and its elevated expression has been associated with poorer prognosis [15, 16]. In our cohort, higher *PKMYT1* expression was associated with the accumulation of chromosomal alterations. This finding aligns with previous studies suggesting a role for *PKMYT1* in maintaining genomic integrity under conditions of replication stress or impaired G1/S checkpoint control [17, 18].

Functional enrichment analyses provided additional insights into the biological context of *PKMYT1* expression in CLL. ORA analysis highlighted processes related to chromatin remodeling, DNA repair, and transcriptional regulation, while enrichment of components such as the centrosome and RNA‐binding functions suggested a link to genome integrity and RNA metabolism. Although GSEA did not yield statistically significant pathways after correction, several top‐ranked gene sets implicated roles in DNA replication, telomere maintenance, and metabolic regulation. Together, these findings suggest potential biological associations that merit further investigation and support the hypothesis that *PKMYT1* deregulation may contribute to transcriptional programs involved in genome maintenance, metabolic reprogramming, and chromatin dynamics in CLL.

In the public dataset, patients with higher *PKMYT1* expression had significantly longer overall survival compared to those with lower expression. It should be noted that survival data were not available for our local cohort, and therefore the prognostic implications of PKMYT1 expression require further validation in independent patient populations. While the underlying mechanisms remain to be clarified, these observations are consistent with the hypothesis that higher *PKMYT1* expression could reflect an adaptive cellular response to DNA damage, potentially restraining cell cycle progression and limiting further genomic instability. Conversely, reduced *PKMYT1* expression may be associated with less effective genomic control, which could contribute to the expansion of aggressive clones. These findings highlight context‐dependent roles of PKMYT1 in CLL and underscore the need for further studies to determine the functional and clinical implications of its expression.

Survival analyses were conducted using public in silico cohorts that include patients exposed to heterogeneous treatment strategies, representing an inherent limitation of this retrospective analysis. Despite this variability, the consistent association between *PKMYT1* expression and overall survival across a large and diverse cohort supports its relevance as a marker of intrinsic disease biology rather than a treatment‐specific effect.

Functional assays further demonstrated that pharmacological inhibition of PKMYT1 in CLL‐derived MEC1 cells significantly reduced cell viability and induced apoptosis, without significantly affecting healthy donor PBMCs. These findings indicate a degree of selectivity toward malignant cells. Previous studies have shown that PKMYT1 inhibition disrupts mitotic progression and sensitizes cells to DNA damage, thereby promoting mitotic collapse, apoptosis, and, in certain contexts, PANoptosis [16, 19].

To further investigate the mechanisms underlying cell death induced by PKMYT1 inhibition, we analyzed the expression of genes associated with distinct cell death pathways in MEC1 cells. Treatment with the PKMYT1 inhibitor resulted in increased expression of apoptosis‐related genes, including *TP53, CASP3,* and *BAK,* consistent with activation of apoptotic signaling. In addition, we observed upregulation of genes involved in inflammatory cell death pathways, such as *CASP1, GSDMD, IL1β, RIPK1, and RIPK3.*

These findings indicate that PKMYT1 inhibition primarily promotes apoptotic cell death, while also inducing transcriptional changes associated with inflammatory cell death pathways. However, whether these transcriptional changes translate into functional activation of pyroptosis or related inflammatory cell death programs remains to be determined. Such changes may reflect cellular stress responses triggered by mitotic disruption and DNA damage signaling following PKMYT1 inhibition.

Several limitations of this study should be acknowledged. The relatively limited size of the local patient cohort restricted the statistical power for certain clinico‐molecular and subgroup analyses, particularly with respect to the evaluation of associations with individual cytogenetic lesions. Nevertheless, the consistency of the observed associations within this cohort, together with the independent validation of the prognostic relevance of PKMYT1 in a larger external dataset, supports the biological relevance of our findings. Future studies in larger, uniformly annotated cohorts will be essential to further refine the clinical and biological significance of *PKMYT1* expression in CLL.

In addition, emerging evidence indicates that clonal hematopoiesis (CH) is frequent in CLL and is associated with adverse outcomes and transcriptional alterations in hematopoietic and immune cells [20, 21]. Future studies should therefore evaluate whether CH contributes to inter‐cohort variability in PKMYT1 expression and modulates PKMYT1‐associated cell cycle and genomic stability pathways.

Collectively, these findings demonstrate that *PKMYT1* is heterogeneously expressed in CLL, correlates with adverse cytogenetic features, and is essential for CLL cell survival. Pharmacological inhibition selectively impairs malignant cells, highlighting PKMYT1 as a promising therapeutic target. These results provide a rationale for further studies to elucidate its mechanistic role and potential clinical application in CLL.

## Author Contributions

Elizabete Cristina Iseke Bispo, Cláudia de Souza Lima Pontes, Jennifer Martins do Nascimento, and Fábio Wilson de Lima Alves performed experiments, analyzed and interpreted data, and drafted the manuscript. Juliana Lott de Carvalho interpreted the data, and drafted the manuscript. Antônio Roberto Lucena‐Araujo obtained patient samples, updated the clinical data, interpreted the data, and drafted the manuscript. Felipe Saldanha‐Araujo conceived and designed the study, interpreted the data, and reviewed the manuscript. The authors read and approved the final manuscript.

## Funding

This work was supported by the Coordenação de Aperfeiçoamento de Pessoal de Nível Superior (CAPES) and Conselho Nacional de Desenvolvimento Científico *e* Tecnológico (CNPq).

## Ethics Statement

The study protocol was approved by the Ethical Committee of the Medical School Hospital of Ribeirão Preto, University of São Paulo, Brazil.

## Consent

Samples were collected after informed consent was obtained from patients.

## Conflicts of Interest

The authors declare no conflicts of interest.

## Data Availability

Data will be made available from the corresponding author on reasonable request.
